# Supplementary material for: Comparative multiomics analysis of cell physiological state after culture in a basket bioreactor
Source: Sci Rep. 2022 Nov 23;12:20161. doi: 10.1038/s41598-022-24687-4 (PMC9686226; doi:10.1038/s41598-022-24687-4)
Supplement: Supplementary file 1 — Supplementary Information 1. [file 41598_2022_24687_MOESM1_ESM.zip › raw data/Transcriptomic raw data/7.AS/2.dasplot/TE1vsTC1/A3SS/14_CCNB3_NW_023666086.1_681386_684308_+@NW_023666086.1_685340_685438_+@NW_023666086.1_685343_685438_+.pdf]

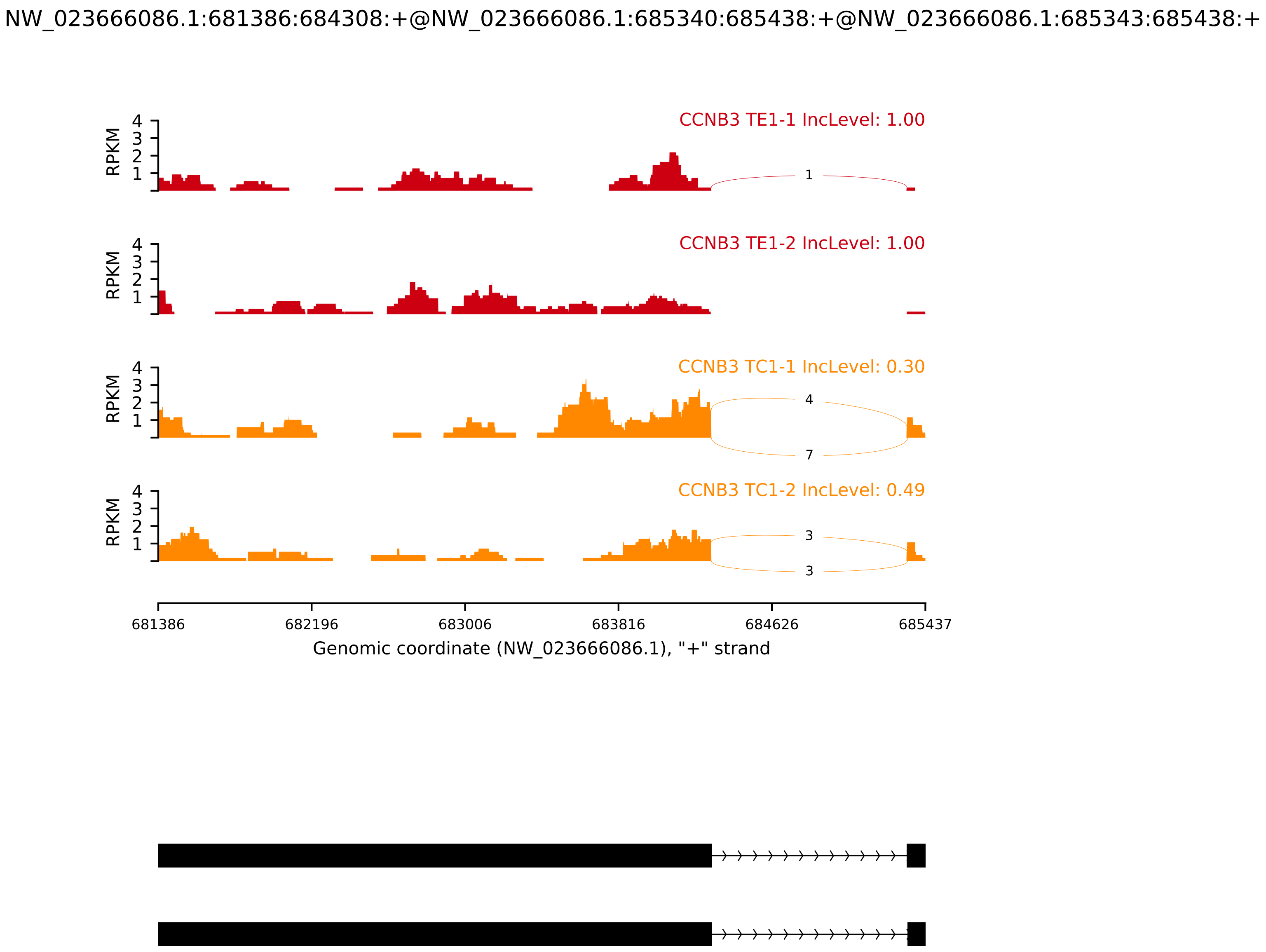

NW_023666086.1:681386:684308:+@NW_023666086.1:685340:685438:+@NW_023666086.1:685343:685438:+
CCNB3 TE1-1 IncLevel: 1.00
CCNB3 TE1-2 IncLevel: 1.00
CCNB3 TC1-1 IncLevel: 0.30
CCNB3 TC1-2 IncLevel: 0.49
RPKM
1
4
7
3
3
681386
682196
683006
683816
684626
685437
Genomic coordinate (NW_023666086.1), "+" strand
